# *Houttuynia cordata* Thunb inhibits the production of pro-inflammatory cytokines through inhibition of the NFκB signaling pathway in HMC-1 human mast cells

**DOI:** 10.3892/mmr.2013.1585

**Published:** 2013-07-11

**Authors:** HEE JOE LEE, HYE-SOOK SEO, GYUNG-JUN KIM, CHAN YONG JEON, JONG HYEONG PARK, BO-HYOUNG JANG, SUN-JU PARK, YONG-CHEOL SHIN, SEONG-GYU KO

**Affiliations:** 1 Department of Oriental Medicine, Kyongwon University, Seongnam, Gyeonggi-Do 461-701, Republic of Korea; 2 Laboratory of Clinical Biology and Pharmacogenomics and Center for Clinical Research and Genomics, Institute of Oriental Medicine, Kyung Hee University, Seoul 130-701, Republic of Korea

**Keywords:** *Houttuynia cordata* Thunb, allergic inflammation, human mast cells, NF-κB, pro-inflammatory cytokine

## Abstract

*Houttuynia cordata* Thunb (HCT) is widely used in oriental medicine as a remedy for inflammation. However, at present there is no explanation for the mechanism by which HCT affects the production of inflammatory cytokines. The current study aimed to determine the effect of an essence extracted from HCT on mast cell-mediated inflammatory responses. Inflammatory cytokine production induced by phorbol myristate acetate (PMA) plus a calcium ionophore, A23187, was measured in the human mast cell line, HMC-1, incubated with various concentrations of HCT. TNF-α, IL-6 and IL-8 secreted protein levels were measured using an ELISA assay. TNF-α, IL-6 and IL-8 mRNA levels were measured using RT-PCR analysis. Nuclear and cytoplasmic proteins were examined by western blot analysis. The NF-κB promoter activity was examined by luciferase assay. It was observed that HCT inhibited PMA plus A23187-induced TNF-α and IL-6 secretion and reduced the mRNA levels of TNF-α, IL-6 and IL-8. It was also noted that HCT suppressed the induction of NF-κB activity, inhibited nuclear translocation of NF-κB and blocked the phosphorylation of IκBα in stimulated HMC-1 cells. It was concluded that HCT is an inhibitor of NF-κB and cytokines blocking mast cell-mediated inflammatory responses. These results indicate that HCT may be used for the treatment of mast cell-derived allergic inflammatory diseases.

## Introduction

Inflammation is part of the complex biological response of vascular tissue to harmful stimuli, including pathogens, damaged cells or irritants (1). This inflammation is associated with cytokines and pro-inflammatory mediators secreted from macrophages. An allergic reaction is the result of an inappropriate immune response triggering inflammation (2). A common example is hay fever, which is caused by a hypersensitive response by skin mast cells to allergens (3). During allergic inflammation, immunoglobulin E (IgE) is produced against allergen infiltration resulting in activation of mast cells, which releases histamine, TNF-α, IL-6, IL-8 and NF-κB (4). Histamine is produced by basophils and by mast cells located in nearby connective tissues (5). Histamine binds to the H1 receptor in target cells to contract gut and bronchus smooth muscle and to increase venular permeability and rheum (6). Histamine increases the permeability of capillaries to white blood cells and various proteins to allow them to engage pathogens in the infected tissue (6). TNF-α is secreted during the allergic mechanism by mast cells, macrophages and T cells. TNF-α causes the expression of adhesion factors to vascular endothelial cells and accumulates white blood cells resulting in an inflammatory response (7,8). IL-6 causes a chronic inflammatory response, activating T cells and producing IgE (9). IL-8 functions as a chemotactic factor for neutrophils, eosinophils and T lymphocytes activating an inflammatory response (10). NF-κB acts as a transcription factor binding to an NF-κB response element located at the promoter of target genes, regulating TNF-α, IL-6 and IL-8 (11,12). The secretion of histamine, TNF-α, IL-6, IL-8 and NF-κB is important in the inflammatory response and appropriate regulation of these molecules may be useful for the treatment of inflammatory disease.

*Houttuynia cordata* Thunb (HCT), a perennial herb, known as ‘E-Sung-Cho’ in Korea, is widely distributed throughout Southeast Asia. Antiviral, anticancer, antileukemic, antioxidant and antiallergic activities of HCT have previously been reported (13–17). HCT may be beneficial for the treatment of mast cell-mediated inflammation (18,19). In addition, HCT extract induces apoptosis via the mitochondrial-dependent pathway in HT-29 human colon adenocarcinoma cells (20).

In the present study, the therapeutic effect of HCT on allergic inflammatory disease was investigated. The levels of TNF-α, IL-6 and IL-8 in HMC-1 human mast cells activated by PMA and A23187 under treatment with HCT were measured. HCT inhibited PMA plus A23187-induced TNF-α and IL-6 secretion and reduced mRNA levels of TNF-α, IL-6 and IL-8. HCT was observed to block the nuclear translocation of NF-κB, inhibiting the phosphorylation of IκBα in stimulated HMC-1 cells. This indicates that HCT inhibits an allergic inflammatory response via inhibition of the NF-κB signaling pathway in HMC-1 human mast cells.

## Materials and methods

### Preparation of HCT ethanol extract

HCT was purchased from Omniherb (Yeongcheon, Republic of Korea). A 100 g ground powder was extracted twice with 80% v/v ethanol using an ultra-sonicator (Branson, Danbury, CT, USA) for 30 min at room temperature. The resulting extract was filtered through a 0.22 μm filter and concentrated to ~100 ml under reducing pressure. The ethanol extract was evaporated at 40°C (Eyela, Tokyo, Japan) and freeze-dried for 72 h (Matsushita, Kadoma, Japan). The powder from the extract was dissolved in DMSO and stored in aliquots at −80°C until further analysis.

### Reagents

Iscove’s modified Dulbecco’s medium (IMDM), fetal bovine serum (FBS), antibiotic-antimycotic and phosphate-buffered saline (PBS) were purchased from Gibco-BRL (Carlsbad, CA, USA). Phorbol 12-myristate 13-acetate (PMA) and A23187 were obtained from Sigma-Aldrich (St. Louis, MO, USA). The MTS assay kit was purchased from Promega Corporation (Madison, WI, USA) and the EZ-western detection kit was obtained from Daeil Lab (Daeil Lab Service Co., Seoul, Korea).

### Antibodies

Anti-human TNF-α antibody, biotinylated anti-human TNF-α antibody and human TNF-α recombinant protein were obtained from R&D Systems (Minneapolis, MN, USA). Anti-human IL-6/IL-8 antibody, biotinylated anti-human IL-6/IL-8 antibody and human IL-6/IL-8 recombinant protein were purchased from BD Biosciences (San Jose, CA, USA). Antibodies against NF-κB, p-IκBα and lamin B were purchased from Santa Cruz Biotechnology, Inc. (Santa Cruz, CA, USA). α-tubulin antibody was from Sigma-Aldrich.

### Cell culture

Human mast cells (HMC-1) were maintained as monolayer cultures in IMDM supplemented with 10% FBS, 100 U/ml of penicillin and 100 μg/ml of streptomycin at 37°C in a humidified incubator under 5% CO_2_ gas.

### MTS assay

HMC-1 cells were seeded at a density of 1×10^6^ cells/well in 24-well plates, pretreated with various concentrations of HCT (0.05–0.4 mg/ml) for 1 h and incubated for 24 h in the absence or presence of PMA (25 nM) plus A23187 (1 μM). Following 24-h incubation, MTS reagents were added to the culture medium prior to the detection of absorbance at 490 nm. Since the absorbance correlates to the viability of cells, the number of cells (% of control) was calculated using the formula: cell number (% of control) = (absorbance of cells treated with CJ or silibinin - absorbance of blank well)/(absorbance of control cells - absorbance of blank well) × 100.

### Measurement of pro-inflammatory cytokines by ELISA (enzyme-linked immunosorbent assay)

HMC-1 cells (1×10^6^) were incubated with various concentrations of HCT (0.05–0.2 mM) for 1 h and treated with PMA plus A23187 for 4 h. To measure pro-inflammatory cytokines, 96-well plates were coated with anti-human TNF-α, IL-6 and IL-8 monoclonal antibodies in 0.1 M sodium carbonate buffer (pH 9.5) and then incubated overnight at 4°C. Following washing, the cells were blocked with 10% FBS in PBS and incubated at room temperature for 1 h. Following additional washing, samples (culture supernatants) were incubated for 2 h at 37°C and washed with PBS containing 0.05% Tween-20 (PBST) and incubated with 0.2 μg/ml biotinylated anti-human TNF-α, IL-6 and IL-8 antibodies at room temperature for 1 h. Incubation with streptavidin-horseradish peroxidase and subsequent treatment with tetramethylbenzidine and hydrogen peroxide substrate in the dark was performed for 30 min together with washing and the reaction was terminated using 2NH_2_SO_4_. Color development was measured using a microplate reader at 450 nm. The inhibition percentage of cytokine production was calculated using the equation: % inhibition = (A – B) × 100/A, where A and B are the cytokine production without and with HCT, respectively.

### RNA extraction and reverse transcription-polymerase chain reaction (RT-PCR)

Total cellular RNA was isolated using an easy-BLUE™ RNA extraction kit (Intron Biotechnology, Seoul, Korea) according to the manufacturer’s instructions. Total RNA (2 μg) was synthesized to cDNA using M-MLV reverse transcriptase (Invitrogen Life Technologies, Carlsbad, CA, USA) according to the manufacturer’s instructions. PCR was conducted in a 20 μl reaction mixture consisting of cDNA template, 10 pmol each gene-specific primer, 10X *Taq* buffer, 2.5 mM dNTP mixture and 1 unit *Taq* DNA polymerase (Takara Korea Biomedical Inc, Seoul, Korea). PCR was performed using the primers: TNF-α, 5′-TGAGCACTGAAAGCATGATCC-3′ and 5′-ATCACTCCAAAGTGCAGCAG-3′; IL-6, 5′-AACC TTTCCAAAGATGGCTGAA-3′ and 5′-CAGGAACTGG ATCAGGACTTT-3′; IL-8, 5′-TCAGTGCATAAAGAC ATACTCC-3′ and 5′-TGGCATCTTCACTGATTCTTG-3′; and GAPDH 5′-CGTCTTCACCACCATGGAGA-3′ and 5′-CGGCCATCACGCCACAGTTT-3′. The sequencing involved thermal cycling at 95°C for 1 min (denaturation), 50°C for 1 min (annealing) and 72°C for 1 min (extension). The products were checked by agarose electrophoresis and analyzed using the ChemiDoc imaging system (Bio-Rad, Hercules, CA, USA).

### Preparation of cytosolic and nuclear protein

Cells were incubated in buffer A [10 mM HEPES (pH 7.9), 10 mM KCl, 1.5 mM MgCl_2_, 0.5 mM dithiothreitol (DTT) and 0.2 mM phenylmethylsulfonyl fluoride (PMSF)]. The cells were incubated on ice for 5 min and centrifuged at 4,000 × g for 5 min. The pellet was then lysed with buffer B [10 mM HEPES (pH 7.9), 10 mM KCl, 1.5 mM MgCl_2_, 0.1% NP-40, 0.5 mM DTT and 0.2 mM PMSF] and centrifuged at 5,000 rpm for 5 min at 4°C. The cytoplasmic proteins were extracted from the supernatant and the pellet was resuspended in buffer C [20 mM HEPES (pH 7.9), 420 mM NaCl, 1.5 mM MgCl_2_, 25% glycerol, 0.2 mM EDTA, 0.5 mM DTT and 0.2 mM PMSF], incubated on ice for 30 min and then centrifuged at 5,000 rpm for 10 min at 4°C. Nuclear proteins were obtained from the supernatant.

### Western blot analysis

An equal amount of protein in total cell extracts was separated by SDS-PAGE. Following electrophoresis, the proteins were transferred to a nitrocellulose membrane (Schleicher & Schuell Bioscience, Dassel, Germany). The membrane was blocked, incubated overnight at 4°C with primary antibodies (anti-NFκB, anti-p-IκBα, anti-lamin B and anti-α-tubulin), washed with PBST (0.1% PBS) and incubated with appropriate HRP-conjugated secondary antibodies at room temperature for 1 h. Immunoreactive protein was developed using an EZ-western detection kit (Daeillab Service Co., Seoul, South Korea).

### Statistical analysis

Data are presented as the mean ± SD. A Student’s t-test was used for single variable comparisons. P<0.05 was considered to indicate a statistically significant difference.

## Results

### Effect of HCT on cell viability in activated mast cells

**Figure 1:**
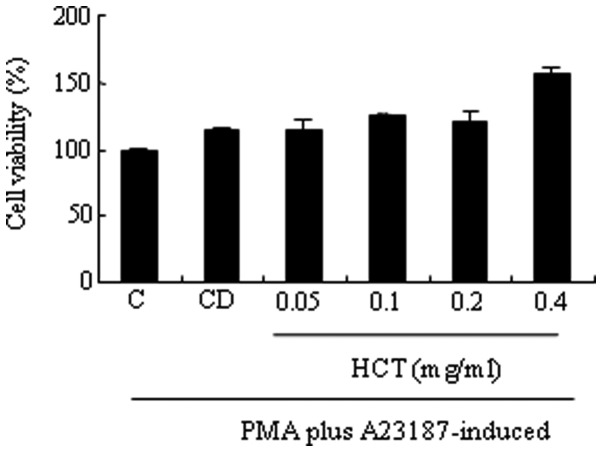
Effect of HCT on cell viability in activated mast cells. HMC-1 cells (1×10^6^ cells/ml) were pretreated with the indicated concentrations of HCT (0.05 −0.4 mg/ml) for 1 h and incubated with PMA and A23187 for 24 h. Cell viability was determined by MTS assay. Data are presented as the mean ± SD of three independent experiments. C, induced control; CD, induced control with DMSO treatment; HCT, *Houttuynia cordata* Thunb.

Mast cells are activated by PMA and A23187 and secrete inflammatory mediators, including histamine, serotonin, hydrolase, heparin and prostaglandin. In the current study, the effect of HCT on cell viability in HMC-1 cells was investigated. It was observed that HCT did not affect cell viability in HMC-1 cells (Fig. 1). HCT failed to decrease histamine release induced by PMA plus A23187 (data not shown).

### Effect of HCT on PMA plus A23187-stimulated TNF-α expression

**Figure 2:**
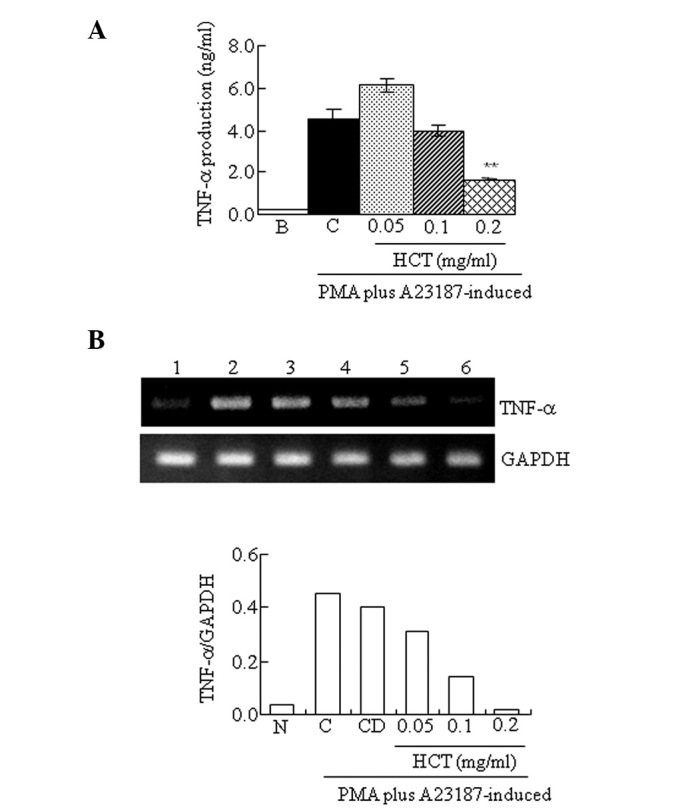
Effect of HCT on PMA plus A23187-stimulated TNF-α expression. HMC-1 cells (1×10^6^ cells/ml) were pre-incubated with various concentrations of HCT (0.05–0.2 mg/ml) for 1 h and treated with PMA plus A23187 for 4 h. (A) TNF-α secreted protein levels in the supernatant were measured by ELISA assay. (B) TNF-α mRNA levels were measured by RT-PCR. Lanes 1, normal cells; 2, control cells; 3, DMSO control cells; 4, HCT (0.05 mg/ml) + PMA plus A23187; 5, HCT (0.1 mg/ml) + PMA plus A23187; 6, HCT (0.2 mg/ml) + PMA plus A23187. Data are presented as the mean ± SD of three independent experiments (^*^P<0.05 and ^**^P<0.01, vs. control). N, no treatment; C, induced control; CD, induced control with DMSO treatment; HCT, *Houttuynia cordata* Thunb; ELISA, enzyme-linked immunosorbent assay; RT-PCR, reverse transcription polymerase chain reaction.

Pro-inflammatory cytokines are important factors of allergic inflammation. Therefore, the production and expression of TNF-α was determined by ELISA or RT-PCR to evaluate the effect of HCT on the pro-inflammatory cytokines. It was observed that HCT significantly decreased TNF-α production induced by PMA plus A23187 (Fig. 2A). In addition, mRNA levels of TNF-α induced by PMA plus A23187 was reduced by HCT treatment (Fig. 2B).

### Effect of HCT on PMA plus A23187-stimulated IL-6 expression

**Figure 3:**
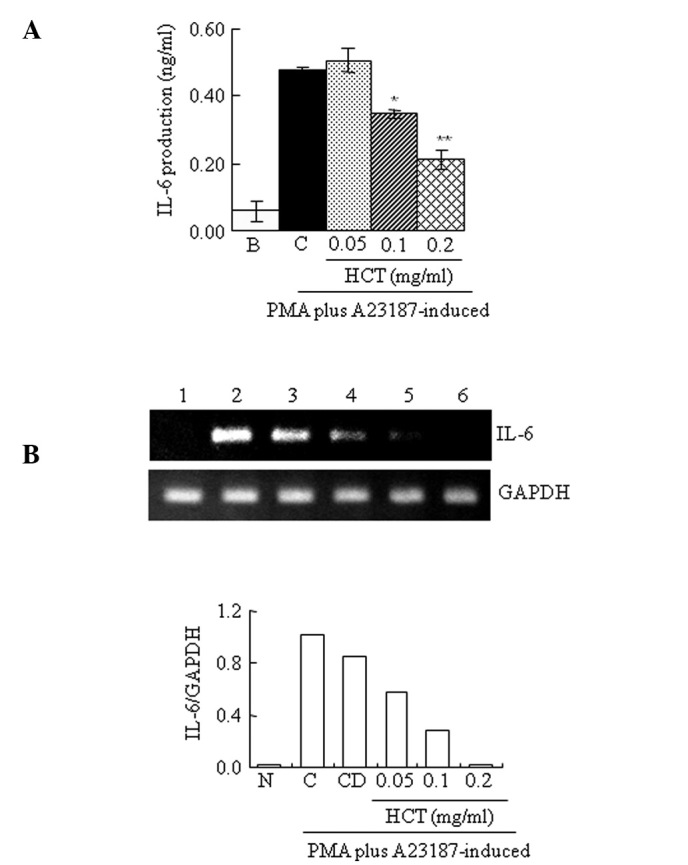
Effect of HCT on PMA plus A23187-stimulated IL-6 expression. HMC-1 cells (1×10^6^ cells/ml) were preincubated with various concentrations of HCT (0.05–0.2 mg/ml) for 1 h and treated with PMA plus A23187 for 4 h (A and B). (A) IL-6 secreted protein levels in the supernatant were measured by ELISA assay. (B) IL-6 mRNA levels were measured by RT-PCR. Lanes 1, normal cells; 2, control cells; 3, DMSO control cells; 4, HCT (0.05 mg/ml) + PMA plus A23187; 5. HCT (0.1 mg/ml) + PMA plus A23187; 6, HCT (0.2 mg/ml) + PMA plus A23187. Data are presented as the mean ± SD of three independent experiments (^*^P<0.05 and ^**^P<0.01 vs. control). N, no treatment; C, induced control; CD, induced control with DMSO treatment; HCT, *Houttuynia cordata* Thunb; ELISA, enzyme-linked immunosorbent assay; RT-PCR, reverse transcription polymerase chain reaction.

The production and expression of IL-6 by ELISA or RT-PCR was determined. HCT significantly decreased IL-6 production induced by PMA plus A23187 (Fig. 3A). In addition, IL-6 mRNA levels induced by PMA plus A23187 were also reduced by HCT treatment (Fig. 3B).

### Effect of HCT on PMA plus A23187-stimulated IL-8 expression

**Figure 4:**
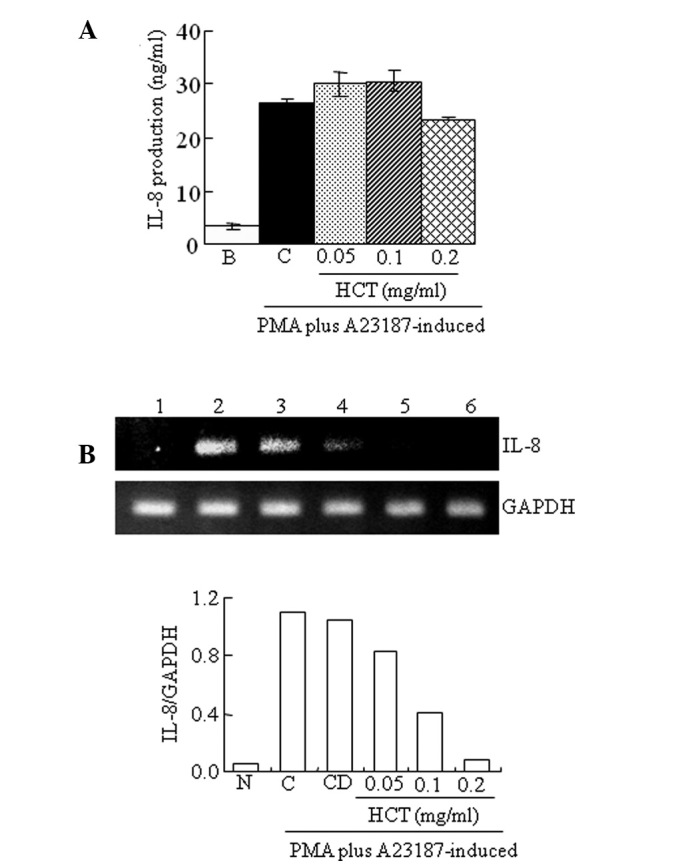
Effect of HCT on PMA plus A23187-stimulated IL-8 expression. HMC-1 cells (1×10^6^ cells/ml) were preincubated with various concentrations of HCT (0.05–0.2 mg/ml) for 1 h and treated with PMA plus A23187 for 4 h (A and B). (A) IL-8 secreted protein levels in the supernatant were measured by ELISA assay. (B) IL-8 mRNA levels were measured by RT-PCR. Lanes 1, normal cells; 2, control cells; 3, DMSO control cells; 4, HCT (0.05 mg/ml) + PMA plus A23187; 5, HCT (0.1 mg/ml) + PMA plus A23187; 6, HCT (0.2 mg/ml) + PMA plus A23187. Data are presented as the mean ± SD of three independent experiments. N, no treatment; C, induced control; CD, induced control with DMSO treatment; HCT, *Houttuynia cordata* Thunb ELISA, enzyme-linked immunosorbent assay; RT-PCR, reverse transcription polymerase chain reaction.

The production and expression of IL-8 was measured by ELISA and RT-PCR. HCT was not found to significantly decrease the production levels of IL-8 induced by PMA plus A23187 (Fig. 4A). However, mRNA levels of IL-8 induced by PMA plus A23187 were reduced by HCT (Fig. 4B).

### Effect of HCT on PMA plus A23187-stimulated NF-κB activation and IκBα phosphorylation

**Figure 5:**
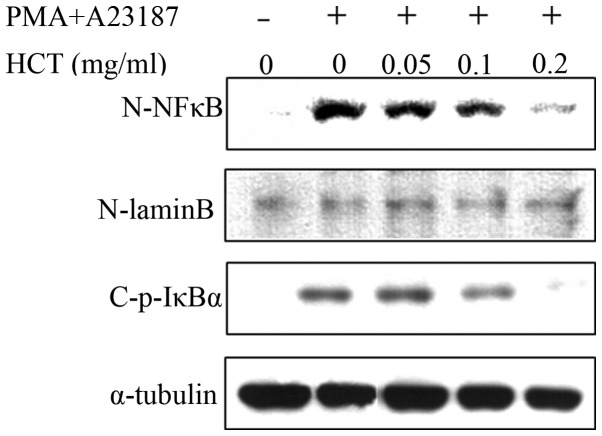
Effect of HCT on PMA plus A23187-stimulated NF-κB activation and IκBα phosphorylation. HMC-1 cells (1×10^6^ cells/ml) were incubated with HCT (0.05–0.2 mg/ml) for 1 h and stimulated with PMA plus A23187 for 2 h. Nuclear and cytoplasmic proteins were isolated by lysis buffer and examined for NF-κB and p-IκBα by western blot analysis. N, nuclear extract; C, cytosol extract; HCT, *Houttuynia cordata* Thunb.

Expression of pro-inflammatory cytokines, including TNF-α, IL-6 and IL-8, is regulated by NF-κB signaling. The effect of HCT on the expression of NF-κB signaling molecules was analyzed and HCT was found to suppress the expression of nuclear NF-κB induced by PMA plus A23187 (Fig. 5). In addition, HCT inhibited the phosphorylation of IκBα in stimulated HMC-1 cells indicating that HCT abrogates the dissociation of IκBα from the NF-κB heterodimer (p65 and p50) to suppress NF-κB signaling (Fig. 5).

## Discussion

Mast cells are major immune cells involved in allergies with versatile physiological functions and are important for allergic activities in response to the stimuli-induced release of histamine (21–23). In addition, the inhibition of TNF-α, IL-6, IL-8 and NF-κB activation has been shown as an indicator of anti-inflammatory events in mast cells (21,24). Although several traditional herbal medicines have been shown to have inhibitory effects on allergy or inflammation using mast cells (25,26), there is little information with regard to the effects of HCT on mast cell-derived allergic inflammation or the molecular mechanisms involved. Therefore, in the present study, we investigated whether HCT has a therapeutic effect on allergic inflammatory disease. Since PMA alone fails to induce the degranulation of mast cells, HMC-1 cells were stimulated by PMA and A23187. Notably, HCT inhibited PMA plus A23187-induced TNF-α and IL-6 secretion and reduced mRNA levels of TNF-α, IL-6 and IL-8. In agreement with the current observations, it was previously reported that HCT inhibits the secretion of TNF-α in the activated macrophage-like cell line, RAW 264.7 (27).

Allergic inflammation is classified into early-phase (or type I immediate hypersensitivity) and late-phase reactions, which result in subsequent chronic allergic inflammation. Release of histamine and other mediators following the crosslinking of the Fc receptor subsequent to the binding of IgE (FcɛRI) to allergen in mast cells is known as early-phase reaction. The IgE-mediated PCA reaction is a sensitive reaction for the detection of small quantities of antibodies and has been used to evaluate the mechanisms of immediate allergy reaction (28). These early-phase responses are followed by a late-phase reaction that typically develops 2–9 h following allergen exposure. In late-phase reactions, the recruitment of leucocytes, including T-cells and neutrophils, is featured (21,29,30). The transition to the late-phase reaction is characterized as the recruitment of leukocytes by upregulating mediators, including IL-8 and TNF-α (21,30). Mast cell-derived IL-8 is hypothesized to activate neurophils in allergic inflammation (31). In a previous study, it was reported that monomeric IgE induces long-lasting IL-8 synthesis in mast cells (32). It was also reported that TNF-α is crucial in the development of late-phase anaphylactic reactions via the PAF-mediated NF-κB-dependent pathway and initiates late-phase allergic inflammation (33). Therefore, the observation of no significant change in histamine release, but significant suppression of inflammatory cytokines, indicates that HCT may have inhibitory effects on allergic inflammation through transition or late-phase reaction.

NF-κB plays a key role in the cellular stress response and in inflammation by controlling the expression of a network of genes, including TNF-α, IL-6 and IL-8. Following infection, microbial pathogens are sensed by the host and activate NF-κB transcription factors via triggering of various sensors, including the toll-like receptors, which are expressed on cells of the innate immune system, macrophages, dendritic cells and mucosal epithelial cells (34,35). NF-κB activation is closely controlled by a pathway that regulates the proteolysis of the inhibitory IκB and IκB-associated proteins. In unstimulated cells, NF-κB dimers are sequestered in the cytoplasm via physical association with NF-κB inhibitory proteins, IκBs (34). Upon stimulation, signal transduction events rapidly lead to the activation of the IκB kinase (IKK) complex, composed of two catalytic subunits (IKKα and IKKβ) and a regulatory subunit, NF-κB essential modulator (34). Activated IKK phosphorylates IκBα, predominantly via the action of IKKβ, triggering its polyubiquitination and proteasomal degradation and inducing the nuclear translocation of associated NF-κB subunits (34). NF-κB subunits bind to specific DNA to induce the transcription of target genes. The current observations indicate that HCT suppresses the nuclear translocation of NF-κB as well as the phosphorylation of IκBα, which may inhibit the expression of proinflammatory cytokines, including TNF-α, IL-6 and IL-8.

Results of the present study suggest that HCT inhibits the production of pro-inflammatory cytokines via inhibition of the NF-κB signaling pathway in HMC-1 human mast cells. We hypothesized that HCT may be a potential therapeutic target for the treatment of allergies and inflammatory diseases.
